# Crystal structure and Hirshfeld surface analysis of 4-bromo­anilinium nitrate

**DOI:** 10.1107/S2056989020006945

**Published:** 2020-05-29

**Authors:** Radhakrishnan Anbarasan, Palaniyasan Eniya, Jeyaperumal Kalyana Sundar, Menberu Mengesha Woldemariam

**Affiliations:** a Materials Science Laboratory, Department of Physics, Periyar University, Salem, India; b Department of Physics, Jimma University, Jimma, Ethiopia

**Keywords:** crystal structure, Hirshfeld surface, hydrogen bonds, aniline

## Abstract

The title compound C_4_H_7_BrN^+^·NO_3_^−^ crystallizes in the monoclinic crystal system with space group *P*2_1_/*c*. In the crystal, π-π stacking inter­actions and strong N—H⋯O and C—H⋯O hydrogen bonds link the cations and anions into layers parallel to the *bc* plane. The O⋯H/H⋯O inter­actions between the cation and anion are the major factor determining the crystal packing.

## Abstract 2

In the crystal, π-π stacking inter­actions and strong N—H⋯O and C—H⋯O hydrogen bonds link the cations and anions into layers parallel to the *bc* plane. The O⋯H/H⋯O inter­actions between the cation and anion are the major factor determining the crystal packing.

## Chemical context   

In recent years, halogenated anilines and their derivatives have been studied extensively for applications as anti­corrosives, anti­bacterials and in non-linear optical systems (Glidewell *et al.*, 2005 ▸; Vivek *et al.*, 2014 ▸). The simplest halogenated aniline readily forms metal/non-metal complexes (Hartmann *et al.*, 1990 ▸). Strong hydrogen bonding, non-covalent bonding and π–π stacking inter­actions are prominent in the supra­molecular arrangements of this mol­ecule. Here, we report the crystal structure of 4-bromo­anilinium nitrate, a salt complex whose structure is closely related to its 4-iodo analogue regarding the hydrogen-bond networks and π–π inter­actions (Fu *et al.*, 2010 ▸) although having significantly different unit-cell parameters.
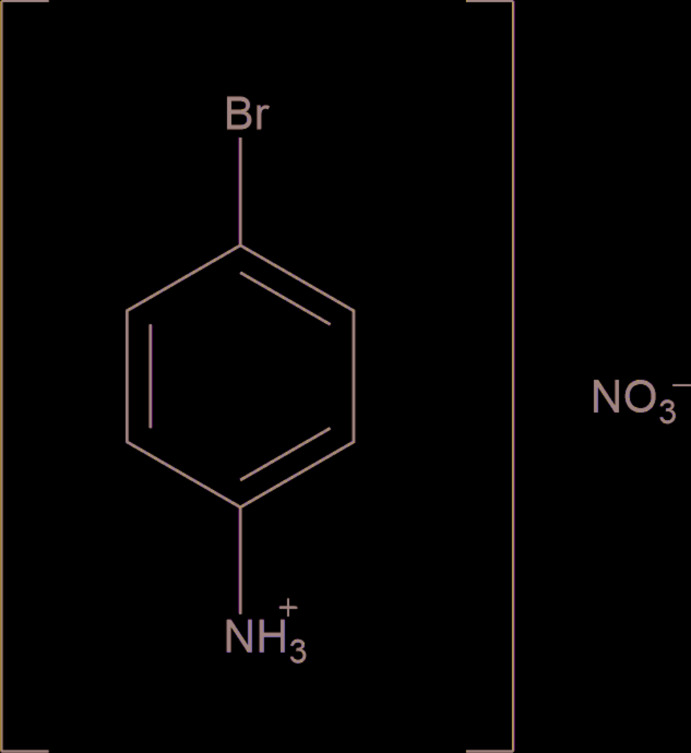


## Structural commentary   

**Figure 1:**
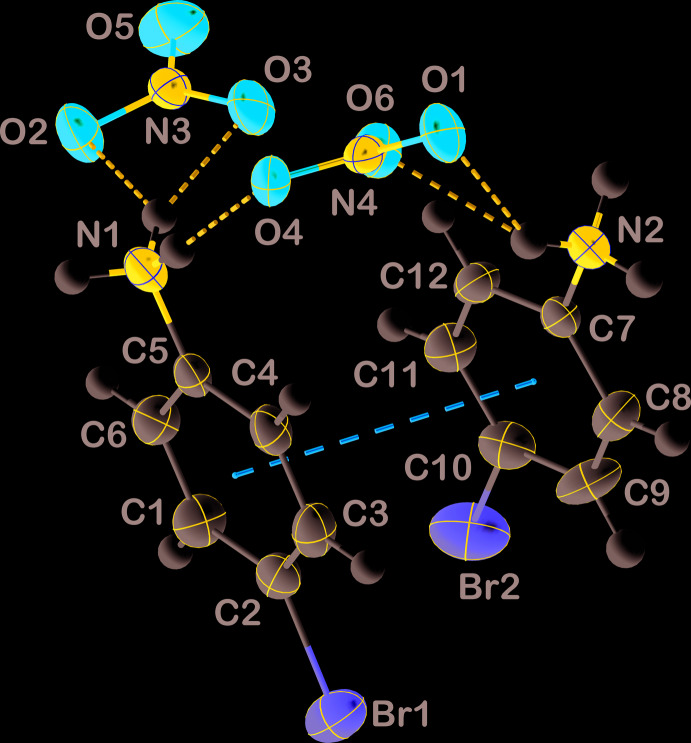
The asymmetric unit with labelling scheme and 50% probability ellipsoids. N—H⋯O hydrogen bonds and π-stacking inter­actions are shown, respectively, by blue and orange dashed lines.

The asymmetric unit consists of two 4-bromo­anilinium cations and two nitrate anions which are associated through N1—H10⋯O4^ii^, N2—H13⋯O3^iv^ and a bifurcated N1—H9⋯O2^i^/N1—H9⋯O3^i^ hydrogen bonds (Fig. 1 ▸). This motif generates a van der Waals contact (O3⋯O6) of 2.980 (4) Å between the two nitrate ions. The phenyl rings in the independent cations extend in the same direction from the pair of anions with a dihedral angle of only 4.8 (2)° between their mean planes and participate in a π–π stacking inter­action with a centroid⋯centroid distance of 3.932 (2) Å. Meanwhile, one cation is rotated with respect to the other so that the Br1—C2⋯C10—Br2 torsion angle is 50.4 **(su?)**°.

## Supra­molecular features   

**Figure 2:**
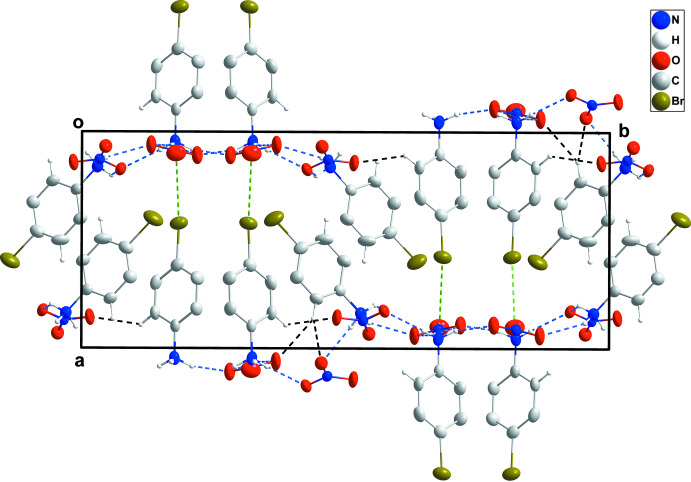
Packing viewed along the *c*-axis direction showing a portion of two coarsely corrugated layers of anions with the cations extending alternately from each side. The N—H⋯O and C—H⋯O hydrogen bonds are shown, respectively, by blue and black dashed lines. The Br⋯O inter­actions are shown by green dashed lines.

In the crystal, the anions are arranged in coarsely corrugated layers parallel to the *bc* plane with the hydrogen-bonded cations protruding from each face in an alternating fashion (Fig. 2 ▸). The cations containing Br1 are perpendicular to the layers and make close Br1⋯O5 contacts of 3.229 (5) Å (0.14 Å less than the sum of the van der Waals radii) with nitrate ions in adjacent layers (Fig. 2 ▸, Table 1 ▸).

## Hirshfeld surface analysis   

**Figure 3:**
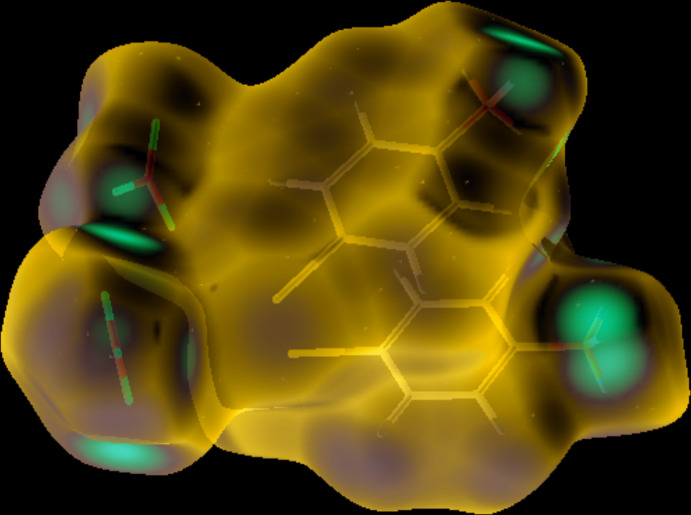
Hirshfeld surface plotted over *d*_norm_.

The inter­molecular inter­actions were investigated qu­anti­tatively and visualized with *Crystal Explorer 3.1* (Wolff *et al.*, 2012 ▸; Spackman *et al.*, 2009 ▸). The *d*_norm_, curvedness and 2D fingerprint plots are depicted in Figs. 3 ▸–5 ▸ ▸, respectively. The red spots on the Hirshfeld surface represent N—H⋯O contacts (Br⋯O contacts are not visible as red spots) while the blue regions correspond to weak inter­actions such as C—H⋯O contacts. The two triangles in the curvedness surface clearly illustrate the π–π stacking inter­actions. The O⋯H/H⋯O (51.4%) inter­actions are the major factor in the crystal packing with H⋯H (15.5%) inter­actions representing the next highest contribution. The percentage contributions of other weak inter­actions are: H⋯Br/Br⋯H (10.3%), C⋯H/H⋯C (9.2%), O⋯Br/Br⋯O (4.1%), Br⋯Br (2.7%), N⋯H/H⋯N (1.7%), O⋯O (1.6%), C⋯C (1.5%), C⋯O/O⋯C (0.8%), N⋯Br/Br⋯N (0.4), C⋯Br/Br⋯C (0.4%), N⋯O/O⋯N (0.3%) and N⋯C/C⋯N (0.1%).

### Database survey   

A search of the Cambridge Structural Database (CSD version 5.41, last update April 2020; Groom *et al.*, 2016 ▸) for the 4-bromo­anilinium cation gave 22 hits excluding metal complexes. Among these, 13 structures have this cation combined with various acid anions including [PO_2_(OH)_2_]^−^ (EBEFAV; Yoshii *et al.*, 2015 ▸; UGISEI; Zhang *et al.*, 2001 ▸; UGISEI01; Yoshii *et al.*, 2015 ▸), [HC_2_O_4_]^−^ (ROBXOY; Radhakrishnan & Jeyaperumal, 2019 ▸), [C_4_H_5_O_6_]^−^ (ROPTEX; Yoshii *et al.*, 2014 ▸) and [*p*-CH_3_C_6_H_4_SO_3_]^−^ (VUCBAY; Sivakumar *et al.*, 2015 ▸). Two more have amide anions [N(SO_2_ *R*)_2_]^−^ [*R* = Me (TAJWOT; Jones *et al.*, 2016 ▸), 4-BrC_6_H_4_ (DOHSOJ; Lozano *et al.*, 2008 ▸)]. The remainder have inorganic anions such as [SiF_6_]^2−^ (PBANIL; Denne *et al.*, 1971 ▸), [PF_6_]^−^ (TUPWUX; Yang & Fu, 2010 ▸) and chloride (TAWRAL; Portalone, 2005 ▸). Additionally, there is an unpublished structure of the title compound (ROCNOP; Anbarasan & Sundar, 2019 ▸) of comparable quality to the present study but without the additional investigations presented here.

## Synthesis and crystallization   

The title salt was synthesized by dissolving analytical grade 4-bromo­aniline and nitric acid in a 1:1 stoichiometric ratio in methanol. The solution was stirred continuously for 2 h. Slow evaporation of this solution at room temperature yielded transparent colourless single crystals of the product.

## Refinement   

Crystal data, data collection and structure refinement details are summarized in Table 2 ▸. The H atoms were positioned geometrically and refined using a riding model: C—H = 0.93 Å with *U*_iso_(H) = 1.2*U*_eq_(C) and N—H = 0.86 Å with *U*_iso_(H) = 1.2*U*_eq_(N). Reflection (100) was obscured by the beam stop and was omitted during the final refinement cycle.

## Supplementary Material

Crystal structure: contains datablock(s) global, I. DOI: 10.1107/S2056989020006945/mw2155sup1.cif

Supporting information file. DOI: 10.1107/S2056989020006945/mw2155Isup2.cml

CCDC reference: 1909800

Additional supporting information:  crystallographic information; 3D view; checkCIF report

## Figures and Tables

**Figure 4:**
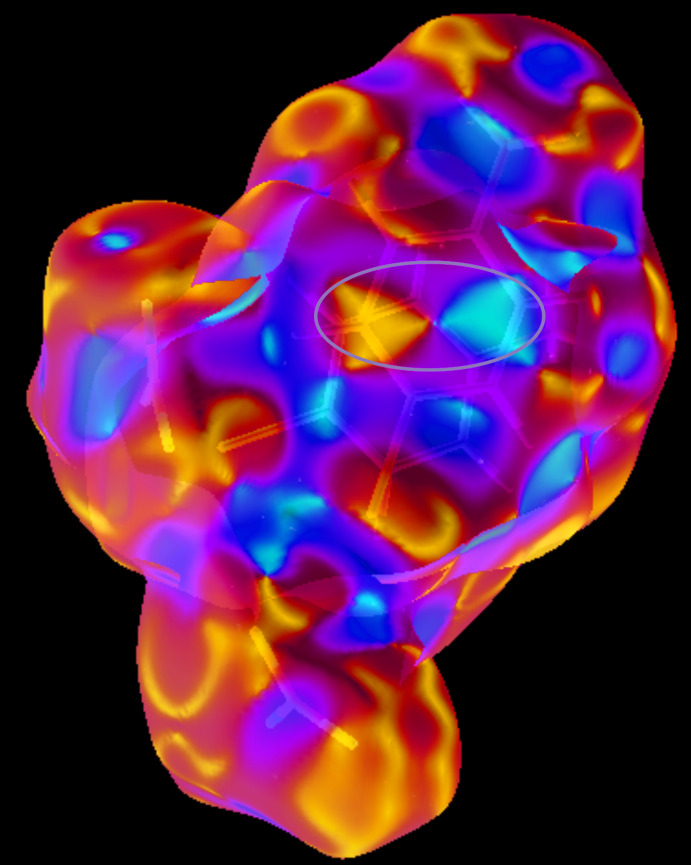
Curvedness surface of the title compound showing the π–π stacking.

**Figure 5:**
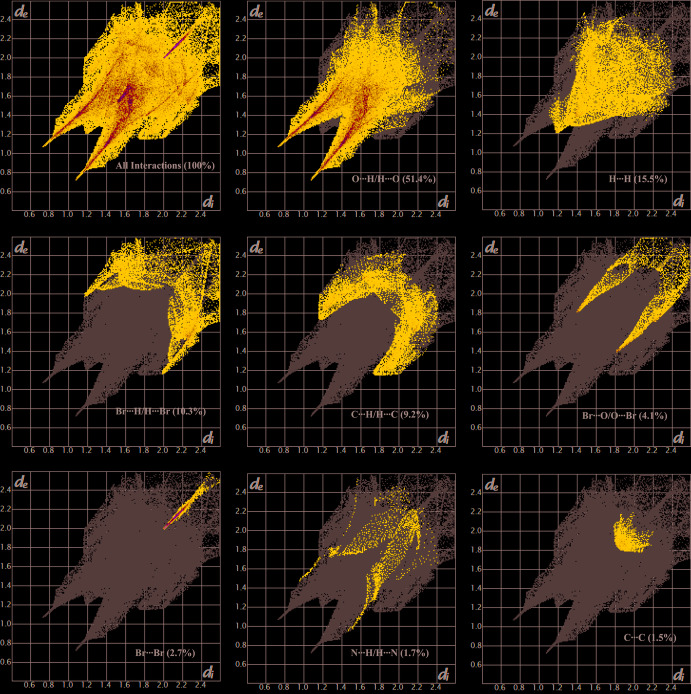
Fingerprint plots for the title compound

**Table 1:** ydrogen-bond geometry (Å, °)

*D*—H⋯*A*	*D*—H	H⋯*A*	*D*⋯*A*	*D*—H⋯*A*
N1—H9⋯O2^i^	0.89	2.19	2.930 (5)	140
N1—H9⋯O3^i^	0.89	2.15	3.002 (5)	160
N1—H10⋯O4^i^	0.89	2.08	2.957 (4)	167
N1—H11⋯O2^ii^	0.89	1.91	2.773 (4)	162
N2—H12⋯O1^i^	0.89	2.59	3.356 (6)	145
N2—H12⋯O6^i^	0.89	2.11	2.827 (5)	137
N2—H12⋯O1^iii^	0.89	2.59	3.158 (5)	122
N2—H13⋯O3^iv^	0.89	2.12	2.774 (5)	130
N2—H13⋯O6^iv^	0.89	2.55	3.345 (5)	149
N2—H14⋯O4^iii^	0.89	2.19	2.831 (5)	129
C4—H3⋯O1^iii^	0.93	2.41	3.129 (5)	134
C12—H8⋯O3^i^	0.93	2.59	3.410 (5)	147
C12—H8⋯O6^i^	0.93	2.58	3.1943 (3)	124

Symmetry codes: (i) 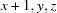; (ii) 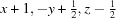; (iii) 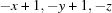; (iv) 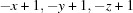.

**Table 2:** Experimental details

Crystal data	
Chemical formula	C_6_H_7_BrN^+^·NO_3_^−^
*M*_r_	235.04
Crystal system, space group	Monoclinic, *P*2_1_/*c*
Temperature (K)	293
*a*, *b*, *c* (Å)	9.7123 (8), 23.4964 (19), 7.6264 (6)
β (°)	97.052 (4)
*V* (Å^3^)	1727.2 (2)
*Z*	8
Radiation type	Mo *K*α
μ (mm^−1^)	4.73
Crystal size (mm)	0.42 × 0.18 × 0.12

Data collection	
Diffractometer	Bruker SMART APEXII CCD
Absorption correction	Multi-scan (*SADABS*; Sheldrick, 1996 ▸)
*T*_min_, *T*_max_	0.374, 0.567
No. of measured, independent and observed [*I* > 2σ(*I*)] reflections	16821, 4609, 2355
*R*_int_	0.058
(sin θ/λ)_max_ (Å^−1^)	0.684

Refinement	
*R*[*F* ^2^ > 2σ(*F* ^2^)], *wR*(*F* ^2^), *S*	0.059, 0.183, 1.02
No. of reflections	4609
No. of parameters	218
H-atom treatment	H-atom parameters constrained
Δρ_max_, Δρ_min_ (e Å^−3^)	0.68, −0.84

Computer programs: *APEX2* and *SAINT* (Bruker, 2004 ▸), *SHELXT* (Sheldrick, 2015*a* ▸), *SHELXL2017* (Sheldrick, 2015*b* ▸), *ORTEP-3 for Windows* and *WinGX* (Farrugia, 2012 ▸), *PLATON* (Spek, 2020 ▸), *Mercury* (Macrae *et al.*, 2020 ▸) and *publCIF* (Westrip, 2010 ▸).

## supplementary crystallographic information

### Crystal data

**Table:**

C_6_H_7_BrN^+^·NO_3_^−^	*F*(000) = 928
*M**_r_* = 235.04	*D*_x_ = 1.808 Mg m^−^^3^
Monoclinic, *P*2_1_/*c*	Mo *K*α radiation, λ = 0.71073 Å
Hall symbol: -P 2ybc	Cell parameters from 4609 reflections
*a* = 9.7123 (8) Å	θ = 2.6–29.1°
*b* = 23.4964 (19) Å	µ = 4.73 mm^−^^1^
*c* = 7.6264 (6) Å	*T* = 293 K
β = 97.052 (4)°	Needle, colorless
*V* = 1727.2 (2) Å^3^	0.42 × 0.18 × 0.12 mm
*Z* = 8	

### Data collection

**Table:**

Bruker SMART APEXII CCD diffractometer	2355 reflections with *I* > 2σ(*I*)
Radiation source: fine-focus sealed tube	*R*_int_ = 0.058
ω and φ scan	θ_max_ = 29.1°, θ_min_ = 2.1°
Absorption correction: multi-scan (SADABS; Sheldrick, 1996)	*h* = −13→11
*T*_min_ = 0.374, *T*_max_ = 0.567	*k* = −29→32
16821 measured reflections	*l* = −8→10
4609 independent reflections	

### Refinement

**Table:**

Refinement on *F*^2^	Hydrogen site location: inferred from neighbouring sites
Least-squares matrix: full	H-atom parameters constrained
*R*[*F*^2^ > 2σ(*F*^2^)] = 0.059	*w* = 1/[σ^2^(*F*_o_^2^) + (0.0927*P*)^2^ + 0.2455*P*] where *P* = (*F*_o_^2^ + 2*F*_c_^2^)/3
*wR*(*F*^2^) = 0.183	(Δ/σ)_max_ < 0.001
*S* = 1.02	Δρ_max_ = 0.68 e Å^−^^3^
4609 reflections	Δρ_min_ = −0.84 e Å^−^^3^
218 parameters	Extinction correction: SHELXL2018/3 (Sheldrick 2015b)
0 restraints	Extinction coefficient: 0.0181 (17)

### Special details

**Table:**

Geometry. All esds (except the esd in the dihedral angle between two l.s. planes) are estimated using the full covariance matrix. The cell esds are taken into account individually in the estimation of esds in distances, angles and torsion angles; correlations between esds in cell parameters are only used when they are defined by crystal symmetry. An approximate (isotropic) treatment of cell esds is used for estimating esds involving l.s. planes.

### Fractional atomic coordinates and isotropic or equivalent isotropic displacement parameters (Å^2^)

**Table:**

	*x*	*y*	*z*	*U*_iso_*/*U*_eq_	
N1	1.0463 (4)	0.32338 (12)	0.2075 (4)	0.0513 (8)	
H10	1.083534	0.351431	0.150589	0.077*	
H9	1.063697	0.329131	0.323465	0.077*	
H11	1.082988	0.290327	0.180092	0.077*	
N2	0.8383 (4)	0.52952 (13)	0.2737 (5)	0.0571 (9)	
H14	0.798745	0.556374	0.202137	0.086*	
H13	0.880154	0.545557	0.371772	0.086*	
H12	0.900523	0.510739	0.219564	0.086*	
N3	0.0880 (3)	0.32409 (14)	0.6621 (5)	0.0481 (8)	
N4	0.1286 (3)	0.46630 (13)	0.1594 (5)	0.0531 (9)	
O2	0.1085 (4)	0.28155 (12)	0.5705 (5)	0.0762 (10)	
O3	0.0604 (4)	0.36877 (12)	0.5772 (5)	0.0891 (12)	
O4	0.1736 (3)	0.42637 (11)	0.0733 (4)	0.0613 (8)	
O5	0.0995 (5)	0.32142 (18)	0.8212 (5)	0.0941 (12)	
O6	0.0681 (3)	0.45499 (12)	0.2920 (4)	0.0656 (8)	
O1	0.1429 (4)	0.51579 (11)	0.1125 (5)	0.0766 (10)	
C1	0.6768 (5)	0.28047 (18)	0.1770 (6)	0.0632 (12)	
H1	0.622297	0.253605	0.225586	0.076*	
C2	0.6166 (5)	0.31894 (18)	0.0536 (5)	0.0549 (11)	
C3	0.6968 (5)	0.35869 (17)	−0.0173 (6)	0.0611 (12)	
H2	0.655897	0.384689	−0.099684	0.073*	
C4	0.8371 (5)	0.36032 (15)	0.0328 (5)	0.0539 (11)	
H3	0.891543	0.387124	−0.016287	0.065*	
C5	0.8970 (4)	0.32216 (14)	0.1558 (5)	0.0449 (9)	
C6	0.8179 (5)	0.28202 (17)	0.2278 (6)	0.0576 (11)	
H4	0.859217	0.256095	0.310167	0.069*	
C7	0.7319 (4)	0.48981 (15)	0.3193 (5)	0.0466 (9)	
C8	0.5964 (5)	0.49725 (19)	0.2493 (6)	0.0638 (12)	
H5	0.571499	0.527478	0.173184	0.077*	
C9	0.4971 (5)	0.4596 (2)	0.2925 (7)	0.0779 (14)	
H6	0.40465	0.463954	0.245794	0.094*	
C10	0.5363 (5)	0.4159 (2)	0.4046 (6)	0.0640 (12)	
C11	0.6718 (5)	0.40804 (19)	0.4725 (6)	0.0607 (11)	
H7	0.696645	0.377689	0.548105	0.073*	
C12	0.7710 (4)	0.44517 (19)	0.4285 (5)	0.0562 (11)	
H8	0.863752	0.439985	0.472598	0.067*	
Br1	0.42315 (6)	0.31590 (3)	−0.02005 (7)	0.0826 (3)	
Br2	0.40126 (7)	0.36433 (3)	0.47100 (8)	0.1017 (3)	

### Atomic displacement parameters (Å^2^)

**Table:**

	*U*^11^	*U*^22^	*U*^33^	*U*^12^	*U*^13^	*U*^23^
N1	0.070 (2)	0.0398 (16)	0.0435 (19)	−0.0049 (16)	0.0034 (17)	−0.0031 (14)
N2	0.056 (2)	0.0473 (19)	0.065 (2)	0.0020 (16)	−0.0038 (17)	−0.0121 (17)
N3	0.0476 (19)	0.0412 (18)	0.054 (2)	−0.0059 (14)	0.0019 (16)	0.0022 (17)
N4	0.0414 (19)	0.0379 (18)	0.075 (3)	−0.0029 (15)	−0.0123 (17)	0.0051 (18)
O2	0.113 (3)	0.0430 (17)	0.072 (2)	−0.0017 (16)	0.010 (2)	−0.0044 (15)
O3	0.119 (3)	0.0353 (16)	0.103 (3)	0.0007 (17)	−0.026 (2)	0.0073 (17)
O4	0.0609 (19)	0.0433 (15)	0.079 (2)	0.0103 (14)	0.0043 (15)	−0.0019 (14)
O5	0.095 (3)	0.140 (3)	0.047 (2)	0.007 (2)	0.0077 (19)	−0.007 (2)
O6	0.067 (2)	0.0597 (18)	0.072 (2)	−0.0003 (15)	0.0160 (17)	0.0087 (16)
O1	0.097 (3)	0.0328 (15)	0.099 (3)	−0.0081 (15)	0.007 (2)	0.0106 (16)
C1	0.068 (3)	0.065 (3)	0.058 (3)	−0.006 (2)	0.012 (2)	0.015 (2)
C2	0.063 (3)	0.060 (3)	0.042 (2)	0.008 (2)	0.0049 (19)	−0.007 (2)
C3	0.084 (4)	0.051 (2)	0.048 (3)	0.013 (2)	0.006 (2)	0.010 (2)
C4	0.073 (3)	0.036 (2)	0.053 (3)	−0.0020 (19)	0.007 (2)	0.0068 (18)
C5	0.062 (3)	0.0344 (18)	0.038 (2)	0.0014 (17)	0.0056 (18)	−0.0034 (16)
C6	0.070 (3)	0.052 (2)	0.050 (2)	−0.002 (2)	0.004 (2)	0.0139 (19)
C7	0.047 (2)	0.047 (2)	0.044 (2)	0.0037 (18)	0.0029 (17)	−0.0105 (18)
C8	0.054 (3)	0.070 (3)	0.066 (3)	0.013 (2)	0.002 (2)	0.007 (2)
C9	0.042 (3)	0.118 (4)	0.071 (3)	0.004 (3)	−0.005 (2)	0.013 (3)
C10	0.060 (3)	0.078 (3)	0.056 (3)	−0.013 (2)	0.014 (2)	−0.007 (2)
C11	0.063 (3)	0.066 (3)	0.054 (3)	−0.002 (2)	0.008 (2)	0.007 (2)
C12	0.048 (2)	0.065 (3)	0.053 (3)	0.009 (2)	−0.005 (2)	−0.001 (2)
Br1	0.0635 (4)	0.1164 (5)	0.0676 (4)	0.0127 (3)	0.0060 (3)	−0.0013 (3)
Br2	0.0876 (5)	0.1390 (6)	0.0825 (5)	−0.0422 (4)	0.0262 (3)	0.0036 (3)

### Geometric parameters (Å, º)

**Table:**

N1—C5	1.456 (5)	C2—Br1	1.895 (5)
N1—H10	0.89	C3—C4	1.369 (6)
N1—H9	0.89	C3—H2	0.93
N1—H11	0.89	C4—C5	1.375 (5)
N2—C7	1.465 (5)	C4—H3	0.93
N2—H14	0.89	C5—C6	1.373 (6)
N2—H13	0.89	C6—H4	0.93
N2—H12	0.89	C7—C12	1.364 (6)
N3—O5	1.207 (5)	C7—C8	1.370 (6)
N3—O3	1.245 (4)	C8—C9	1.379 (7)
N3—O2	1.249 (4)	C8—H5	0.93
N4—O1	1.229 (4)	C9—C10	1.360 (7)
N4—O4	1.254 (4)	C9—H6	0.93
N4—O6	1.258 (4)	C10—C11	1.366 (6)
C1—C6	1.378 (7)	C10—Br2	1.900 (4)
C1—C2	1.382 (6)	C11—C12	1.372 (6)
C1—H1	0.93	C11—H7	0.93
C2—C3	1.369 (6)	C12—H8	0.93
			
C5—N1—H10	109.5	C3—C4—C5	119.7 (4)
C5—N1—H9	109.5	C3—C4—H3	120.2
H10—N1—H9	109.5	C5—C4—H3	120.2
C5—N1—H11	109.5	C6—C5—C4	120.7 (4)
H10—N1—H11	109.5	C6—C5—N1	119.5 (3)
H9—N1—H11	109.5	C4—C5—N1	119.8 (4)
C7—N2—H14	109.5	C5—C6—C1	119.4 (4)
C7—N2—H13	109.5	C5—C6—H4	120.3
H14—N2—H13	109.5	C1—C6—H4	120.3
C7—N2—H12	109.5	C12—C7—C8	121.2 (4)
H14—N2—H12	109.5	C12—C7—N2	118.9 (4)
H13—N2—H12	109.5	C8—C7—N2	119.9 (4)
O5—N3—O3	123.7 (4)	C7—C8—C9	119.5 (4)
O5—N3—O2	121.3 (4)	C7—C8—H5	120.3
O3—N3—O2	115.0 (4)	C9—C8—H5	120.3
O1—N4—O4	119.8 (4)	C10—C9—C8	119.0 (4)
O1—N4—O6	120.9 (4)	C10—C9—H6	120.5
O4—N4—O6	119.3 (3)	C8—C9—H6	120.5
C6—C1—C2	119.8 (4)	C9—C10—C11	121.5 (4)
C6—C1—H1	120.1	C9—C10—Br2	120.0 (4)
C2—C1—H1	120.1	C11—C10—Br2	118.5 (4)
C3—C2—C1	120.1 (5)	C10—C11—C12	119.6 (4)
C3—C2—Br1	120.0 (3)	C10—C11—H7	120.2
C1—C2—Br1	119.9 (4)	C12—C11—H7	120.2
C2—C3—C4	120.3 (4)	C7—C12—C11	119.2 (4)
C2—C3—H2	119.9	C7—C12—H8	120.4
C4—C3—H2	119.9	C11—C12—H8	120.4
			
C6—C1—C2—C3	−0.4 (7)	C12—C7—C8—C9	1.2 (7)
C6—C1—C2—Br1	178.7 (3)	N2—C7—C8—C9	179.8 (4)
C1—C2—C3—C4	0.4 (6)	C7—C8—C9—C10	0.2 (7)
Br1—C2—C3—C4	−178.7 (3)	C8—C9—C10—C11	−1.1 (8)
C2—C3—C4—C5	−0.6 (6)	C8—C9—C10—Br2	178.5 (4)
C3—C4—C5—C6	0.7 (6)	C9—C10—C11—C12	0.6 (7)
C3—C4—C5—N1	179.2 (3)	Br2—C10—C11—C12	−179.1 (3)
C4—C5—C6—C1	−0.6 (6)	C8—C7—C12—C11	−1.8 (6)
N1—C5—C6—C1	−179.2 (4)	N2—C7—C12—C11	179.6 (4)
C2—C1—C6—C5	0.4 (7)	C10—C11—C12—C7	0.9 (6)

### Hydrogen-bond geometry (Å, º)

**Table:**

*D*—H···*A*	*D*—H	H···*A*	*D*···*A*	*D*—H···*A*
N1—H9···O2^i^	0.89	2.19	2.930 (5)	140
N1—H9···O3^i^	0.89	2.15	3.002 (5)	160
N1—H10···O4^i^	0.89	2.08	2.957 (4)	167
N1—H11···O2^ii^	0.89	1.91	2.773 (4)	162
N2—H12···O1^i^	0.89	2.59	3.356 (6)	145
N2—H12···O6^i^	0.89	2.11	2.827 (5)	137
N2—H12···O1^iii^	0.89	2.59	3.158 (5)	122
N2—H13···O3^iv^	0.89	2.12	2.774 (5)	130
N2—H13···O6^iv^	0.89	2.55	3.345 (5)	149
N2—H14···O4^iii^	0.89	2.19	2.831 (5)	129
C4—H3···O1^iii^	0.93	2.41	3.129 (5)	134
C12—H8···O3^i^	0.93	2.59	3.410 (5)	147
C12—H8···O6^i^	0.93	2.58	3.1943 (3)	124

Symmetry codes: (i) *x*+1, *y*, *z*; (ii) *x*+1, −*y*+1/2, *z*−1/2; (iii) −*x*+1, −*y*+1, −*z*; (iv) −*x*+1, −*y*+1, −*z*+1.

## References

[bb1] Anbarasan, R. & Sundar, J. K. (2019). CSD Communication (refcode ROCNOP). CCDC, Cambridge, England.

[bb2] Bruker (2004). *APEX2* and *SAINT*. Bruker AXS Inc., Madison, Wisconsin, USA.

[bb3] Denne, W. A., Mathieson, A. & Mackay, M. F. (1971). *J. Cryst. Mol. Struct.* **1**, 55–62.

[bb4] Farrugia, L. J. (2012). *J. Appl. Cryst.* **45**, 849–854.

[bb5] Fu, X. (2010). *Acta Cryst.* E**66**, o1326. 10.1107/S1600536810016740 PMC2979466 21579417

[bb6] Glidewell, C., Low, J. N., Skakle, J. M. S. & Wardell, J. L. (2005). *Acta Cryst.* C**61**, o276–o280. 10.1107/S0108270105007286 15876714

[bb7] Groom, C. R., Bruno, I. J., Lightfoot, M. P. & Ward, S. C. (2016). *Acta Cryst.* B**72**, 171–179. 10.1107/S2052520616003954 PMC4822653 27048719

[bb8] Hartmann, J., Dou, S.-Q. & Weiss, A. (1990). *Berichte der Bunsengesellschaft für physikalische Chemie*, **94**, 1110–1121.

[bb9] Jones, P. G., Blaschette, A. & Moers, O. (2016). CSD Communication (refcode TAJWOT). CCDC, Cambridge, England.

[bb10] Lozano, V., Freytag, M., Jones, P. G. & Blaschette, A. (2008). *Z. Naturforsch. B*, **63**, 954–962.

[bb11] Macrae, C. F., Sovago, I., Cottrell, S. J., Galek, P. T. A., McCabe, P., Pidcock, E., Platings, M., Shields, G. P., Stevens, J. S., Towler, M. & Wood, P. A. (2020). *J. Appl. Cryst.* **53**, 226–235. 10.1107/S1600576719014092 PMC6998782 32047413

[bb12] Portalone, G. (2005). *Acta Cryst.* E**61**, o3083–o3085.

[bb13] Radhakrishnan, A. & Jeyaperumal, K. S. (2019). CSD Communication (refcode ROBXOY). CCDC, Cambridge, England.

[bb14] Sheldrick, G. M. (1996). *SADABS*.

[bb15] Sheldrick, G. M. (2015*a*). *Acta Cryst.* A**71**, 3–8.

[bb16] Sheldrick, G. M. (2015*b*). *Acta Cryst.* C**71**, 3–8.

[bb17] Sivakumar, P. K., Kumar, M. K., Kumar, R. M., Chakkaravarthi, G. & Kanagadurai, R. (2015). *Acta Cryst.* E**71**, o163–o164. 10.1107/S2056989015002686 PMC4350755 25844228

[bb18] Spackman, M. A. & Jayatilaka, D. (2009). *CrystEngComm*, **11**, 19–32.

[bb19] Spek, A. L. (2020). *Acta Cryst.* E**76**, 1–11. 10.1107/S2056989019016244 PMC6944088 31921444

[bb20] Vivek, P. & Murugakoothan, P. (2014). *Appl. Phys. A*, **115**, 1139–1146.

[bb21] Westrip, S. P. (2010). *J. Appl. Cryst.* **43**, 920–925.

[bb22] Wolff, S. K., Grimwood, D. J., McKinnon, J. J., Turner, M. J., Jayatilaka, D. & Spackman, M. A. (2012). *Crystal Explorer*. University of Western Australia.

[bb23] Yang, Y. & Fu, X. (2010). *Acta Cryst.* E**66**, o1430. 10.1107/S1600536810018088 PMC2979586 21579506

[bb24] Yoshii, Y., Hoshino, N., Takeda, T. & Akutagawa, T. (2015). *J. Phys. Chem. C*, **119**, 20845–20854.

[bb25] Yoshii, Y., Hoshino, N., Takeda, T., Moritomo, H., Kawamata, J., Nakamura, T. & Akutagawa, T. (2014). *Chem. Eur. J.* **20**, 16279–16285. 10.1002/chem.201404043 25308219

[bb26] Zhang, B.-G., Gou, S.-H., Duan, C.-Y. & You, X.-Z. (2001). *Wuhan Dax. Xuebao, Zir. Kex.* **47**, 425–427.

